# Molecular Phylogenetics and Temporal Diversification in the Genus *Aeromonas* Based on the Sequences of Five Housekeeping Genes

**DOI:** 10.1371/journal.pone.0088805

**Published:** 2014-02-20

**Authors:** J. Gaspar Lorén, Maribel Farfán, M. Carmen Fusté

**Affiliations:** 1 Departament de Microbiologia i Parasitologia Sanitàries, Facultat de Farmàcia, Universitat de Barcelona, Barcelona, Spain; 2 Institut de Recerca de la Biodiversitat (IRBio), Universitat de Barcelona, Barcelona, Spain; Auburn University, United States of America

## Abstract

Several approaches have been developed to estimate both the relative and absolute rates of speciation and extinction within clades based on molecular phylogenetic reconstructions of evolutionary relationships, according to an underlying model of diversification. However, the macroevolutionary models established for eukaryotes have scarcely been used with prokaryotes. We have investigated the rate and pattern of cladogenesis in the genus *Aeromonas* (γ-*Proteobacteria*, *Proteobacteria*, Bacteria) using the sequences of five housekeeping genes and an uncorrelated relaxed-clock approach. To our knowledge, until now this analysis has never been applied to all the species described in a bacterial genus and thus opens up the possibility of establishing models of speciation from sequence data commonly used in phylogenetic studies of prokaryotes. Our results suggest that the genus *Aeromonas* began to diverge between 248 and 266 million years ago, exhibiting a constant divergence rate through the Phanerozoic, which could be described as a pure birth process.

## Introduction

Speciation is a central topic in evolutionary science and has been the focus of an enormous amount of research, especially during the last 20 years [1]–[3]. Traditionally, the speciation models and the speciation and extinction rates in a population were determined by analysing the fossil record data, but this is not available for all species, is restricted to the last 600 million years and, in the case of prokaryotes, is scarce and confined to very few taxa. The recent expansion of molecular phylogenetics has provided a useful approach to overcoming this problem.

As tools such as DNA sequencing, genomics and proteomics become feasible for larger samples, it has been possible to analyse diversification patterns from molecular data. Phylogenetic trees inferred from molecular sequences, particularly those including all the living species in a higher taxonomic group, provide an indirect record of speciation events that have led to present day species [1].

Since Nee et al. [4] proposed a method to estimate both speciation and extinction rates of a lineage from phylogenies reconstructed from contemporary taxa, several other methods mainly based on birth-death models have been developed [5]–[7]. In the simplest of these models, the birth and death rates of lineages remain constant through time. However, rates of species origination and extinction can vary over time during evolutionary radiations and among lineages [8], [9]. Therefore, several authors have developed methods to estimate changes in diversification rates through time and across lineages from phylogenetic data of extant species [4], [10], [11]. All these methods have potential applications in the study of speciation and extinction processes in organisms with few or no existent fossil records, such as prokaryotes, although a major problem is the difficulty in estimating divergence times. Phylogenetic trees derived from DNA sequences only contain information about the relative timing of reconstructed speciation events (i.e. branch lengths of these trees represent the evolution rate multiplied by the elapsed time).

Since the seminal papers of Zuckerkandl and Pauling [12] and Kimura [13] molecular dating has been based on the molecular clock hypothesis of a constant chronological rate of sequence change [14]. This approach has been regularly challenged by results obtained using datasets from a variety of organisms, ranging from bacteria to primates, which show considerable departures from clocklike evolution and constant rate variation among lineages, and it has become clear that the strict molecular clock hypothesis is not biologically realistic [15]. This implies that although it is possible to infer phylogenies from molecular sequences, it is not possible to estimate molecular rates or divergence times, because the individual contribution of each one to molecular evolution cannot be separated [15]–[17].

Models that take into account rate variation across lineages have been proposed in order to obtain better estimates of divergence time: the so called ‘relaxed molecular clock models’. These models represent an intermediate position between the ‘strict’ molecular clock hypothesis and the unconstrained models (that do not distinguish times from rates). They include local clocks [18], and nonparametric approaches such as penalized likelihood [19], Bayesian parametric models [15], [20] and a maximum likelihood approach with discrete rate variation (MLA) recently developed by Paradis [21]. Among these, the Bayesian methods allow the use of prior distributions, which quantify the uncertainty in the values of the unknown model parameters before the data are calculated and offer the opportunity of exploring a wide diversity of alternative models, each of which corresponds to specific assumptions concerning the shape of the tree and the way the rate of substitution changes with time [15].

Although prokaryotes represent the majority of living organisms, and dominated the first 80% of the history of life, the macroevolutionary models established for eukaryotes have been scarcely applied to them [22], and the origin of a bacterial lineage or the way in which it has diversified remains largely unexplored. There are only a few references in the literature about bacterial diversification [22]–[24], and in no case has the reported analysis been as complete as those published on higher organisms.

Among the challenges associated with the study of macroevolutionary patterns in microorganisms, one of the most significant is to determine if the diversification rate is constant or varies over time. The limited studies on bacterial macroevolution have been mainly based on pathogenic bacteria, in which diversification rates seem to vary over time [24]. Controversially, the very few studies on free-living or symbiotic bacteria suggest a constant rate of diversification [22], [23].

The genus *Aeromonas* Stanier 1943 [25] is a γ-*Proteobacteria* (*Proteobacteria*, Bacteria) that comprises a group of Gram-negative, rod-shaped bacteria, which are autochthonous to aquatic environments worldwide and are usual microbiota (as well as primary or secondary pathogens) of fish, amphibians and other animals. Some species, mainly *A. caviae*, *A. hydrophila* and *A. veronii* bv. Sobria, are opportunistic pathogens of humans [26]. Hence, *Aeromonas* constitutes a perfect scenario to study the diversification processes in bacteria due to the huge variety of habitats from which its species can be isolated and its combination of free-living bacteria and host-associated strains.

At present a combination of phenotypic, population genetics and phylogenetic studies constitute the best theoretical and practical approach to delineate bacterial and archaeal species [27], which are defined on the basis of phenotypic properties and whole-genome DNA-DNA hybridization (DDH). Each species must have unique phenotypic properties and exhibit more than 70% DDH among strains and 5°C or lower difference of the thermal denaturation midpoint of DNA-DNA heteroduplexes (ΔTm). Studies using both DDH and 16S rRNA gene sequence data illustrate that if two strains show less than 97% of 16S rRNA gene sequence similarity, they are considered separate species [28], [29]. More recent studies have shown that the 70% cut-off point corresponds to 95% of average nucleotide identity (ANI) of the whole genome and 69% of the conserved DNA between strains. With the analysis restricted to the protein-coding portion of the genome, 70% DDH corresponds to 85% ANI or 79% conserved genes [30], [31]. Traditionally, the *Aeromonas* taxonomy has been based on a phenotypical characterization, although some uncertainties have persisted, even after the analysis of a large number of characteristics. To date, most *Aeromonas* species have been taxonomically resolved by phenotypical, molecular and phylogenetic studies. This approach is being widely used in microbial molecular systematics as well as in the phylogenetic analyses of eukaryotic organisms.

We performed a phylogenetic analysis of the genus *Aeromonas* based on the sequences of five housekeeping genes applying Maximum Likelihood (ML) and Bayesian reconstructions and calculated the absolute divergence time by means of Bayesian and Maximum Likelihood Approach (MLA) methods, using the divergence time of *Escherichia coli* and *Salmonella enterica* serovar Typhimurium estimated by Ochman and Wilson [32], [33] as the calibration point. Molecular dating and macro evolutionary birth-death models were used to determine the temporal pattern of lineage diversification and significant changes in diversification rates were estimated using models with constant and variable diversification rates [11], [34]. We evaluated the significance of the gamma statistic, the tree shape and the degree of imbalance as well as the recently developed hypothesis-testing framework that accounts for the possibility that some lineages have not been sampled [35]–[37]. Finally, we discuss our results in light of data reported for macro- and microorganisms, the specific biological characteristics of prokaryotes and current knowledge of macro-diversity through geological time.

## Materials and Methods

### Data Set

A collection of 37 strains belonging to the genus *Aeromonas* was analyzed, including all species and subspecies recognized to date, and several strains considered synonymous or that have been reclassified. We used only one sequence for each species because the inclusion of more strains of the same species would artificially inflate the number of branching events toward the tip of the trees, producing misleading results [38]. For the analysis that needed outgroup rooting, *Escherichia coli* K12 (GenBank accession number NC000913) and *Salmonella enterica* serovar Typhimurium LT2 (GenBank accession number AE006468) were chosen because, despite belonging to another taxonomic group, they are closely related with *Aeromonas*. Five genes under stabilizing selection for encoded metabolic functions (housekeeping genes), widely used in the phylogeny of *Aeromonas* (*cpn60*, *dnaJ*, *gyrB*, *mdh* and *rpoD*), were selected for the analysis [39]–[43]. The nucleotide sequences of these genes were determined in our laboratory according to methods previously described or obtained from the GenBank database. All taxa and GenBank accession numbers of the sequences included in this study are listed in the Online Table S1.

### Alignment and Phylogenetic Analysis

Sequence data were translated aligned using Clustal X according to the system default parameters and translated back to obtain the nucleotide alignments. The sequences were concatenated with the DAMBE program [44] to be used in posterior analysis. DnaSP software (v.5.10, [45]) was applied to determine the DNA polymorphism data. The best fit models of sequence evolution were implemented according to the Akaike Information Criterion (AIC) scores for substitution models evaluated using jModeltest (v.0.1.1, [46], http://darwin.uvigo.es/software/jmodeltest.html).

Phylogenetic relationships were assessed using Maximum Likelihood (ML) and Bayesian inference. ML analysis was performed with PhyML (v.3.0, [47]). ML tree support was evaluated with 500 bootstrap replicates. The tree from PhyML output was obtained from the website http://www.atgc-montpellier.fr/phyml/and visualized using MEGA (v.5, [48]). The aligned matrix and the ML tree generated in this study are available in TreeBASE (www.treebase.org) Study Accession URL: http://purl.org/phylo/treebase/phylows/study/TB2:S13056.

Both Bayesian reconstruction of phylogeny and molecular dating were determined using BEAST (v.1.6.2, [49]). We performed three independent separate Markov Chain Monte Carlo analyses (MCMC) of 10 million generations each, sampling every thousandth generation. In each case, we used an uncorrelated lognormal relaxed-clock model, with a Yule prior on the tree, a GTR+I+G as a substitution model, the default priors for the relaxed clock parameter and a randomly generated starting tree. The resulting log files were monitored for convergence with the CODA package [50]. Traceplots and effective sampling sizes (EESs) were determined using Tracer (v.1.5, [51]). ESS greater than 200 suggests that MCMC chains were run long enough to obtain a valid estimate of the parameters.

The three BEAST runs were combined using Tracer after a burn-in of 10% of generations and used to estimate the posterior distribution of topologies, the divergence times and other parameter values. Node ages and lower bounds of the 95% highest posterior density intervals for divergence times were calculated using TreeAnnotator (v.1.5.4, http://beast.bio.ed.ac.uk/TreeAnnotator) and visualized using FigTree (v.1.3.1, http://beast.bio.ed.ac.uk/FigTree).

In order to determine the maximum credibility tree, we used the 10,000 posterior trees obtained in each run. After discarding the first 5,000 in each case, we sampled 3,000 trees at random. These were combined and from the 9,000 trees obtained we determined the maximum clade credibility tree with a posterior probability limit of 0.5. The outgroup (*E. coli* and *S. enterica*) was pruned.

### Divergent Time Estimations

To test if the sequences evolved in a clock-like manner, we used a clock and a non clock model analysis implemented in BASEML (part of the PAML4 package, [52]). The likelihood values obtained for both models were then compared by a likelihood ratio test (LRT), with LRT = 2 (L_clock_ – L_no clock_) and assuming that this statistic was distributed as a χ^2^ with n–2 degrees of freedom, where n is the number of taxa in our data set. As the LRT test rejected the strict clock model, relative branching times were estimated using two different approaches: a maximum likelihood with discrete rate variation (MLA) implemented in the R package *ape* v. 3.0–7 [10] and the Bayesian uncorrelated relaxed-clock analysis (Bayes) implemented in BEAST. For the Bayesian analysis, the absolute divergence times were calculated indirectly using the estimated divergence time between *E. coli* and *S. enterica* (140±20 Ma, [32]). As we used an indirect approach to determine the calibration point, we applied a normal distribution as the prior with a mean of 140 Ma and a standard deviation of 10 Ma, providing a prior range of 116.7–163.2 Ma (99% CI). For the maximum likelihood approach (MLA), the ML tree obtained with PhyML was converted to ultrametric using the *chronos* function [21] of the R package *ape* and dated according to the divergence time estimated from *E. coli* and *S. enterica*. This method assumes a discrete variation in rates, so it is possible to categorize branches according to the different rates (we used 10 branch categories). The method calculates the contribution of each branch to the maximum likelihood function by summing the contribution of each rate category weighted by its frequency [21]. We also performed a simulation to find the optimal smoothing parameter (lambda) corresponding to our data, with smoothing values ranging from 10^−6^ to 10^6^ with increments of 10, using the same *chronos* function.

### *Aeromonas* Diversification Rates

To visualize the temporal pattern of lineage diversification in *Aeromonas* we performed a semilogarithm lineage-through-time plot (LTT plot) with the R packages *ape* and LASER (v.2.3; [11], [53] using the ultrametric trees obtained from our data by MLA and Bayesian methods.

Three different approaches were used to test significant changes in the diversification rates. First, we used LASER to perform a ML analysis to test whether diversification rates have changed over time, contrasting the likelihood values of the data (branching times derived from the trees) under models with constant diversification rates with those obtained under models where rates varied through time, to detect temporal shifts in diversification in the phylogeny. In this analysis, we included two models with a constant speciation rate (λ), a pure birth model with a λ >0 and extinction rate μ = 0 (the Yule process), and a birth-death model with λ >0 and μ >0. Three rate-variable models were also considered: two multi-rate variants of the Yule model and Yule 2-rates that assume the existence of two breakpoints in time, in which λ reaches different values before and after the breakpoints; a logistic density-dependent speciation rate model (DDL) under which λ at time t is modelled as λ(t) = λ_0_ (1–N_t_/K) where λ_0_ is the initial speciation rate, N_t_ the number of lineages at time t and K is a constant analogous to the carrying capacity parameter of population ecology; and an exponential density-dependent model (DDX), in which λ(t) = λ_0_ N_t_^−x^, and x controls the value of the rate change in the number of lineages at any point in time. The ΔAIC_RC_ test was used to statistically evaluate the fit of the temporal pattern of lineage diversification in *Aeromonas* to this set of rate-constant and rate-variable models. It was computed as ΔAIC_RC_ = AIC_RC_ – AIC_RV_ where AIC_RC_ is the AIC score for the best-fit rate-constant model of diversification, and AIC_RV_ is the AIC for the best-fit rate-variable model under consideration. A negative ΔAIC_RC_ value suggests that data are best approximated by a rate-constant model of diversification [11], [34].

Secondly, we determined the gamma (γ) statistic [36] as implemented in LASER from the Bayesian and MLA chronograms. This statistic compares the relative position of nodes in a phylogeny to those expected under a constant diversification rate model, in which the statistic follows a standard normal distribution. The significance of γ was also determined by calculating its value in 5,000 simulated phylogenies obtained under the Yule model of speciation with the same size and diversification rate as those obtained from our data. Phylogenies were simulated using the R package TreeSim [54]. Broadly, positive values of γ signify that nodes are closer to the tips than what is expected under the constant rate model, while negative values might indicate an apparent deceleration [37]. The analysis of diversification shifts using the γ statistic may produce results biased toward negative values if all taxa of the group are not included in the phylogeny. To overcome this problem, Pybus and Harvey [36] developed the Monte Carlo Constant Rates test (MCCR test), which conducts γ statistic analysis for incompletely sampled phylogenies and estimates the significance of negative values of γ, taking into account a possible undersampling in the phylogeny [37]. Although in this analysis we have included all the known species and subspecies of the genus *Aeromonas*, new species are likely to be described in the future. Therefore, to compute the significance of our γ estimates (Bayesian and MLA), we have conducted the MCCR test implemented in LASER. 5,000 phylogenies were simulated with various clade sizes under the Yule model diversification process. Taxa were randomly pruned from the tree to mimic incomplete sampling. The null distribution of the γ statistic was then calculated from these phylogenies and compared with the observed empirical γ.

The shape of a phylogenetic tree contains useful information about the process of cladogenesis. Measuring the degree of imbalance or asymmetry of a tree topology may provide support for the hypothesis that species have the same or different potential for speciation. Under the Yule model, each extant species is equally likely to split into two daughter-species. Several statistics have been introduced for assessing the level of asymmetry of a tree. These statistics are often used to test whether the tree topology differs significantly from a null model with a constant rate of speciation, commonly the Yule model, in which each external branch on a rooted tree has an equal probability of splitting [55]. We have applied two tests that measure the balance of the tree: Colless’ index (*I_c_*) [56] and the number of cherries (*C_n_*) [35].

Among the various alternative statistics that measure the balance of phylogenetic trees, *I_c_* is simple, intuitive, and powerful [55], [57]. It computes the sum of absolute values |L–R| at each node of the tree, where L and R are the size of the left and right daughter clades, respectively. This sum is often renormalized by dividing it by its maximum possible value: (n–1)(n–2)/2, n being the number of leaves of a tree. Therefore, this statistic varies between 0 and 1: for a completely balanced tree, *I_c_* equals zero, while a value of one indicates that the tree is completely imbalanced.

The mean and standard deviation of Colless’ index under the null hypothesis of Yule trees have been computed by Blum et al. [58]. We applied a Colless test based on a Monte Carlo estimate of the *P* value from quantiles of replicate trees generated under the Yule model. We used the R package apTreeshape (v.1, [59]) to compute the normalized *I_c_* in order to check the balance of our phylogenies and compare them with the Yule model.

McKenzie and Steel [35] considered a simple and easily computed statistic for evaluating the tree shape: the number of cherries of a tree (*C_n_*, where n is the number of tips in a tree). They defined a cherry as a pair of leaves that are adjacent to a common ancestor node. The authors analyzed the distribution of this statistic under the Yule model, and calculated the mean (E [*C_n_*] = n/3) and variance (Var [*C_n_*] = 2n/45) of the number of cherries. We used the R package *ape* to determine the number of cherries in our phylogenies and then compared the values with those calculated theoretically and by simulation, in order to test the rate of homogeneity across clades.

The remaining analyses and graphs not specified in this section were done in the R environment (R Development Core Team, [60], http://www.r-project.org/) using the packages base, *ape*, LASER and TreeSim.

## Results

### Data Set and Phylogenetic Analysis

**Figure 1:**
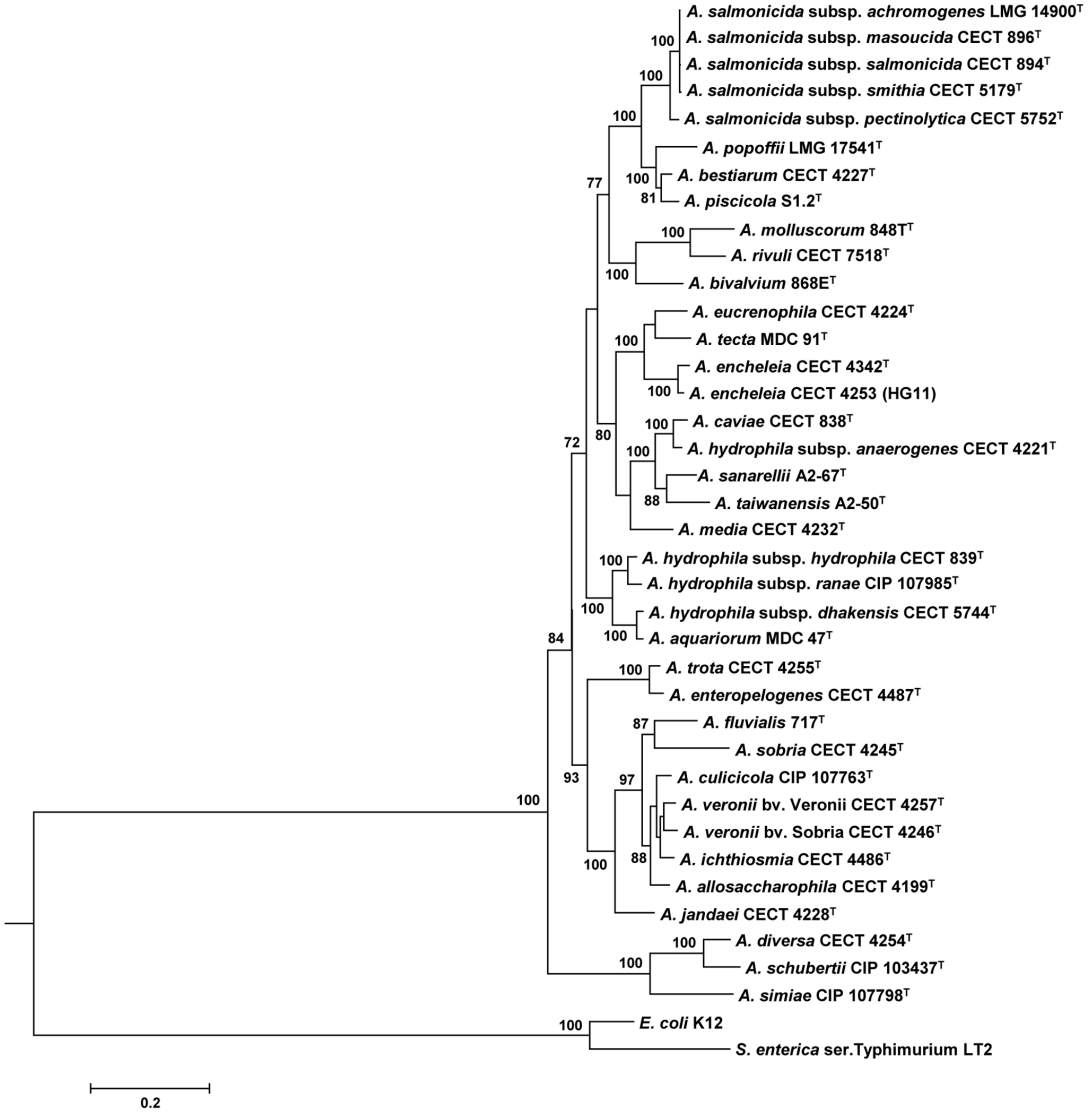
*Aeromonas* species maximum likelihood phylogenetic tree. *E.coli* and *S. enterica* were used as the outgroup. Nodes supported by bootstrap values ≥70% are indicated. The scale bar represents 20% sequence divergence.

The analysis involved 37 *Aeromonas* strains in which we determined the gene sequence of five housekeeping genes *(cpn*60, *dnaJ*, *gyrB*, *mdh* and *rpoD*). The number of total positions in the concatenated sequences was 3,774 bp, with a proportion of 2,140 invariable sites and 1,634 polymorphic sites, 1,423 of which were parsimony informative. The average identity among the concatenated sequences of the *Aeromonas* species was 90.0% (ranging from 86.0 to 91.2%), while the average identity between the *Aeromonas* species and the outgroups *E. coli* and *S. enterica* was 73.9% and 73.2%, respectively. All positions containing gaps and missing data were eliminated in the construction of the different trees. The best model selected for the concatenated sequences was the General Time Reversible (GTR) using a discrete Gamma distribution and a fraction of invariable sites (GTR+G+I). The mean distance between species was 10.0% ±2.6. The uncorrected divergence with the same concatenated sequence between *E. coli* and *Salmonella* was 11.2%. The mean distance between *Aeromonas* species and *E. coli* was 26.1% ±0.5, and 26.8% ±0.5 between *Aeromonas* species and *Salmonella*. Figure 1 shows the *Aeromonas* ML phylogeny, in which the bootstrap support was higher than 70% for the majority of clades. We also performed other phylogenetic reconstructions (Bayesian, Neighbor-Joining, Minimum-Evolution), which gave identical topologies (data not shown).

### Divergence Time Estimations

To determine if our sequences evolved in a clock-like manner we applied two models, a clock and a non-clock model analysis implemented in BASEML. The results obtained shown that our data do not support an assumption of a strict molecular clock model (χ^2^ = 405.3; d.f. = 35; *P*<<0.001). As the LRT test rejected the strict clock model, we used the Bayesian and MLA approaches to estimate the relative branching times.

**Figure 2:**
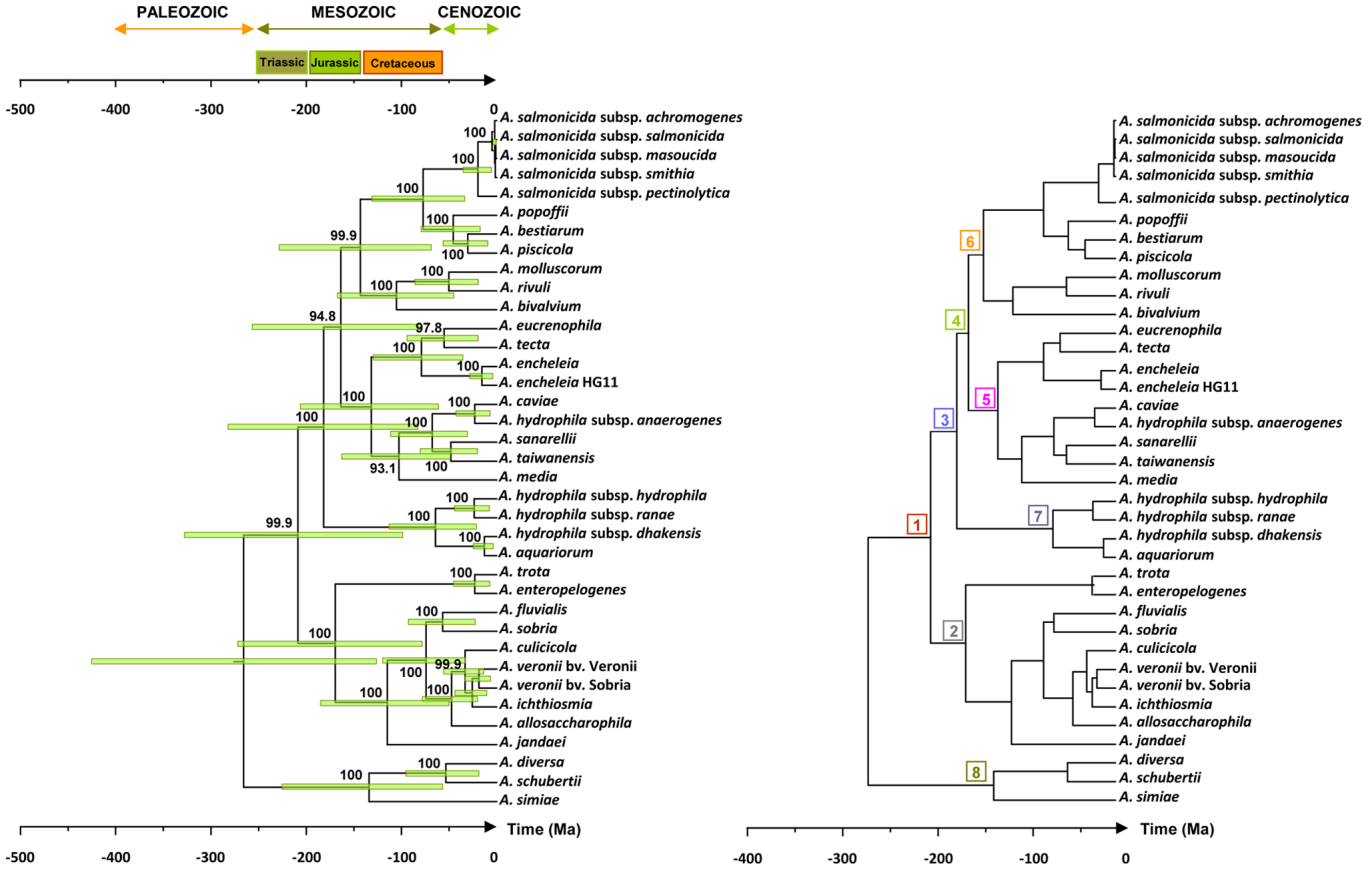
Molecular chronograms of *Aeromonas*. Chronograms were estimated using Bayesian (left) and MLA (right) methods. Bars at the node intersections in the Bayesian chronogram indicate 95% Highest Posterior Density (HPD). Bayesian posterior probability values are shown at the nodes. Time scale is indicated in Mega Annum (Ma). Major *Aeromonas* species clades are indicated by numbers within squares in the MLA chronogram.

Figure 2 shows the chronograms obtained by Bayesian and MLA analyses. In both trees, all clades were coincident and well supported. Age estimates from Bayesian and MLA chronograms were similar. Table 1 show the divergence times for the major clades obtained in the chronograms. Our estimates for the origin of the genus *Aeromonas* ranged from about 248 to 266 Ma ago, depending on the chronogram construction method. Our molecular data suggest that *Aeromonas* diversification began approximately 250 Ma ago and was completed during the last 50 Ma (Fig. 2).

**Table 1:** Age estimates (Ma) of all strains and the major clades of *Aeromonas.*

		Bayesian			MLA		
Clade^a^	N	Age	Lower95%HPD	Upper95%HPD	Age	Lower95% CI	Upper95% CI
All	37	265.7	126.3	425.3	247.8	223.7	270.5
1	34	208.9	98.9	327.7	185.0	167.0	202.3
3	24	181.9	82.8	281.9	159.1	143.7	174.0
4	20	163.6	79.3	256.8	146.6	132.5	160.4
6	11	143.1	68.7	228.6	132.5	119.7	145.0
2	10	169.7	78.4	271.6	150.3	135.6	164.3
5	9	131.6	61.2	206.7	118.0	106.4	129.2
7	4	64.4	21.4	113.1	62.4	56.4	68.1
8	3	134.0	56.7	225.4	122.2	110.3	133.3

a clade numbers appear in the MLA chronogram in Figure 2.

Abbreviations: N, clade size; HPD, the highest posterior density interval; CI, confidence interval.

### *Aeromonas* Diversification Rates

**Figure 3:**
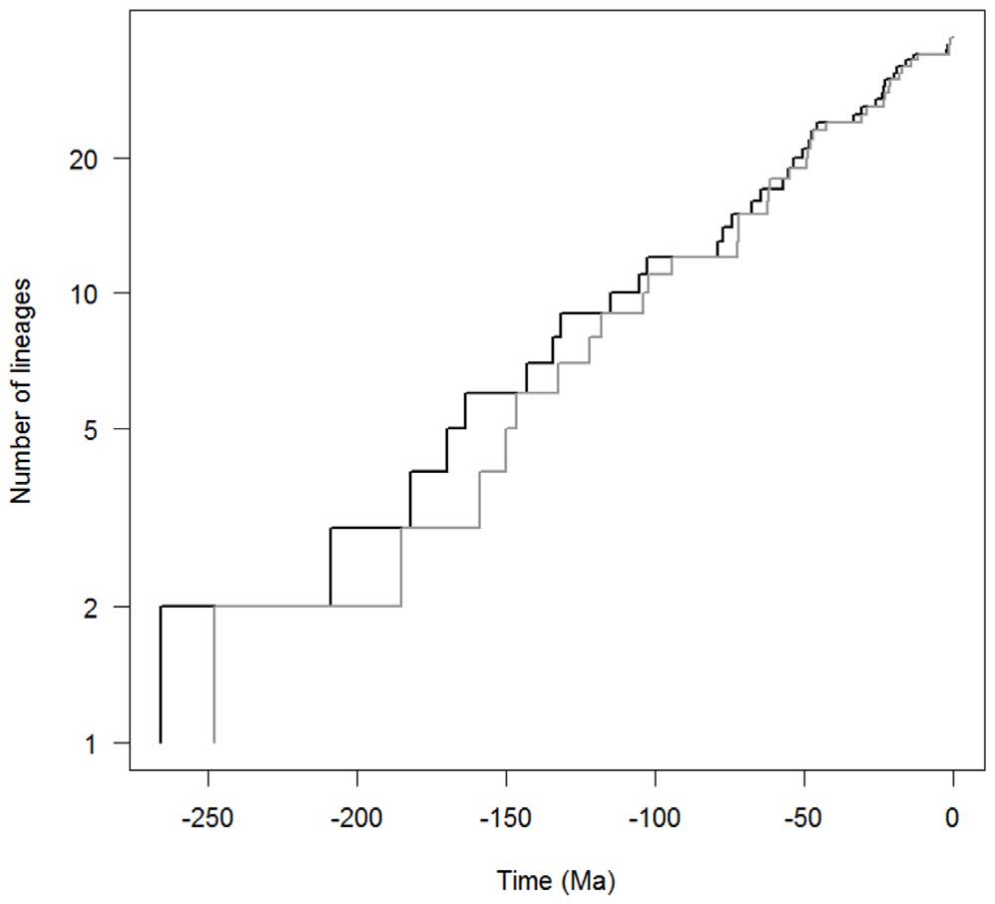
LTT plots for the genus *Aeromonas*. Log-lineage-through-time (LTT) plot for the genus *Aeromonas* based on Bayes (dark line) and MLA (grey line) approaches.

The expected number of lineages versus time (LTT plots) is widely used to characterize clade diversification as a function of time [4], [61]. The semi-logarithmic LTT plots derived from the Bayesian and MLA chronograms are shown in Figure 3. A simple inspection of these plots reveals that the *Aeromonas* lineage accumulation through time appears as a straight line with stochastic fluctuations, which suggests a constant diversification rate. Moreover, the plots do not exhibit any abrupt changes that would suggest the existence of a clear “push of the past” or a clear “pull of the present”, which would be expected if there had been a relatively high extinction rate [61].

To confirm if the diversification rate is really constant or has changed over time, we used maximum likelihood to fit the branching times derived from our chronograms to a variety of diversification models [34], [53]. As suggested by Rabosky, we calculated the significance of ΔAIC_RC_ for the set of analyzed models by simulating 5,000 phylogenies of the same size and diversification rate as those obtained from our data under the Yule model and calculating the *P* value from the resulting distributions. As can be seen in Table 2, in both analyses (Bayesian and MLA) the null hypothesis of a Yule model cannot be rejected to a level of significance of α = 0.05. In concordance with the differences in divergence times obtained in the Bayesian (265.7 Ma) and MLA (247.7 Ma) chronograms, we also observed a slight difference in the diversification rates obtained with the Bayesian (λ_Bayes = _0.0119) and MLA analysis (λ_MLA_ = 0.0129). In conclusion, these results suggest that a Yule model of diversification provides the best fit for our data (Table 2).

**Table 2:** Fit of alternative diversity models to LTT plots derived from Bayes and MLA chronograms.

	AIC^a^							ΔAIC_RC_ test^b^			
Method	Yule	Birth-Death	Bestconstantmodel	DDL	DDX	Yule 2rates	Bestvariablemodel	ΔAIC_RC_	*P* value^e^	Bestmodel	λ (ML) ± se^f^
Bayes	190.64	192.64	Yule	193.06	192.60	193.28^c^	DDX	−1.2668	0.6359	Yule	0.0119±0.0020
MLA	185.06	187.07	Yule	187.15	186.98	187.17^d^	DDX	−1.9119	0.8928	Yule	0.0129±0.0012

a Akaike Information Criterium.

b ΔAIC_RC_ test. See text for details.

c breakpoint at 13 Ma ago.

d breakpoint at 1.3 Ma ago.

e *P* value obtained by simulation (5,000 iterations). See text for additional explanation of simulations.

f standard error.

**Figure 4:**
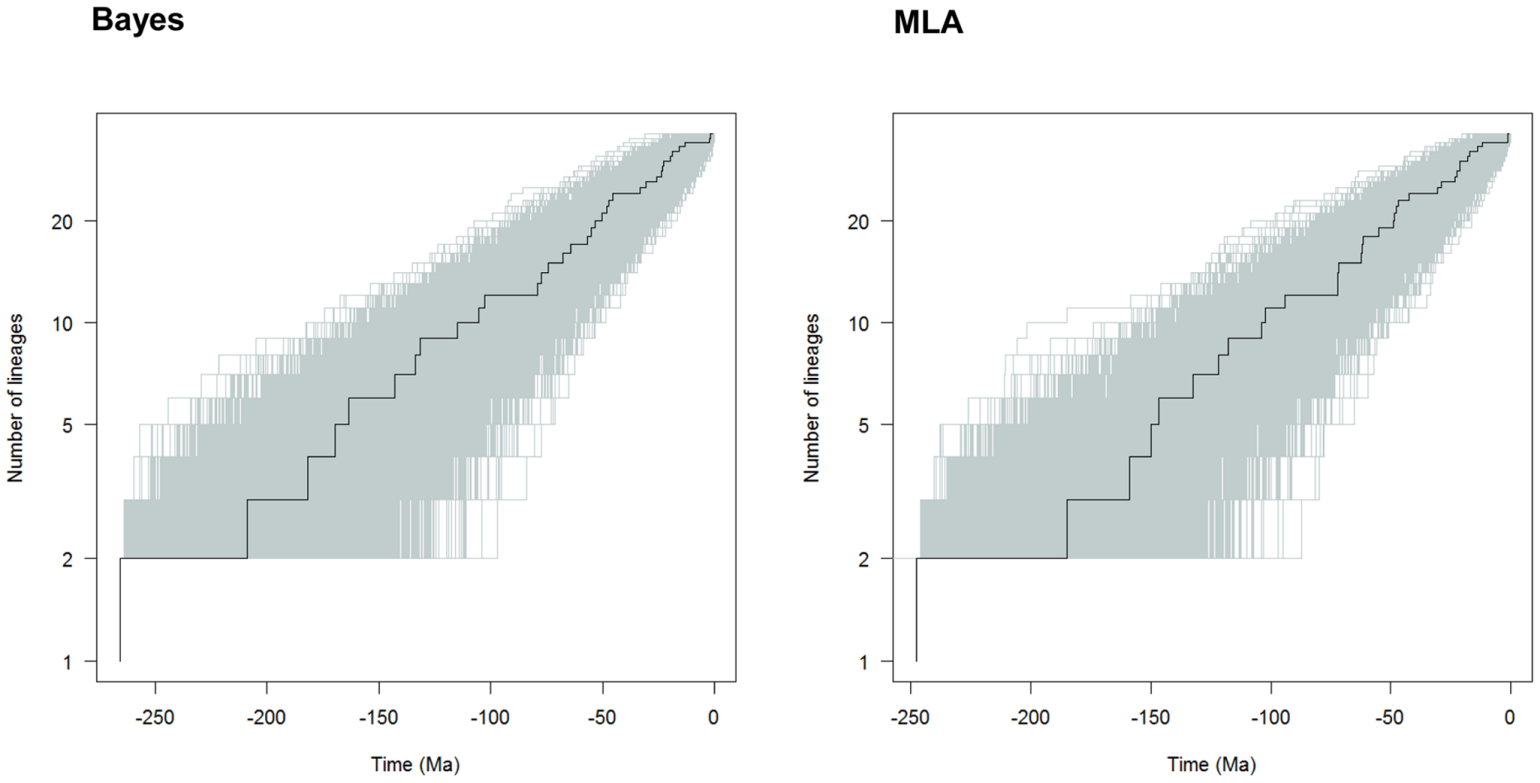
Testing departure of the empirical chronograms of *Aeromonas* from a constant rate diversification model. Dark lines represent the LTT plots obtained for empirical Bayesian (left) and MLA (right) *Aeromonas* phylogenies, while grey lines correspond to the LTT plots of 5,000 simulated phylogenies. In both cases, the root was rescaled to the time to the most recent common ancestor.

To corroborate this conclusion, we compared our LTT plots with those obtained from 5,000 simulated trees under a Yule process with the same size and diversification rate, rescaling the root to the time to the most recent common ancestor. Figure 4 shows that the *Aeromonas* LTT plot (dark line) lays within the range of the simulated phylogenies (grey lines).

To verify that there is not an increase in the diversification rate toward the present in our LTT plots (pull of the present), we fit a Yule model to temporal windows that include the last 200, 100, 50 and 25 million years using the R package LASER. The results obtained supplied respective diversification rates of 0.0122, 0.0116, 0.0113 and 0.0123 for the Bayes chronogram and 0.0134, 0.0124, 0.0129 and 0.0137 for the MLA chronogram. Additionally, to corroborate the absence of a decrease in the diversification rate deep in the phylogeny (push of the past), we fit the Yule model to the first 100 and 150 million years, the results obtained being 0.0119 and 0.0127 for the Bayes and 0.0118 and 0.0139 for the MLA chronograms. In this case, we were unable to fit the model to a smaller window due to the low number of speciation events in this period of time.

**Figure 5:**
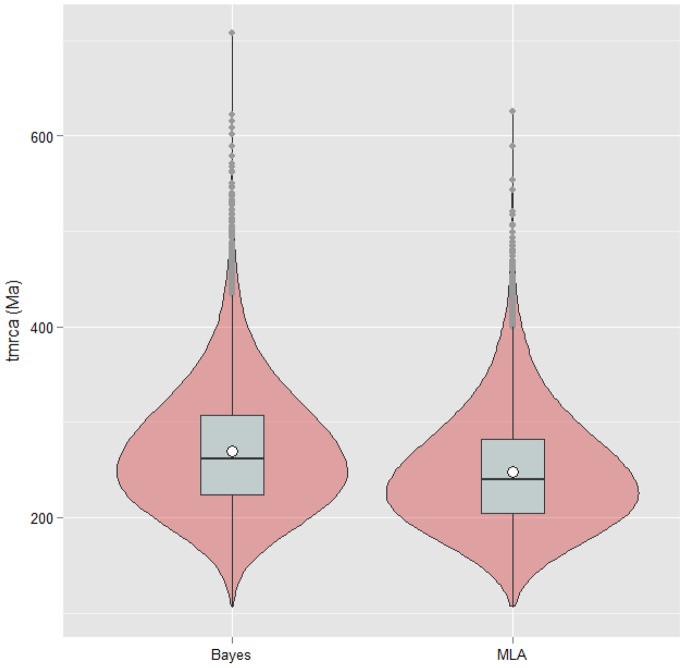
Violin plots of the tmrca distribution for Bayes and MLA simulated trees. The plots show the kernel density estimation of the data (mirrored curves) with a box and whiskers plot overlaid. The plots are scaled so each one has the same total area. The internal box plots indicate the range (whiskers), interquartile range (boxes), median (horizontal black lines) and mean (circles) of the data. The grey points on the plot tails indicate the outliers. See text for more details.

To study the distribution of time to the most recent ancestor (tmrca), we simulated 9,000 phylogenies under the Yule model assuming that *Aeromonas* is a monophyletic group and follows this diversification pattern. The only constraints were the diversification rates (0.0119 for Bayes and 0.0129 for MLA) and the number of species (37) of our phylogenies. Figure 5 shows the distribution of tmrca for Bayes and MLA simulated phylogenies (BSP and MLASP, respectively). The tmrca mean obtained from BSP was 268.3 Ma (95% CI: 164.1–422.5) and 246.7 Ma from MLASP (95% CI: 148.9–385.8 Ma). The divergence times calculated for the genus *Aeromonas* (265.7 Ma from Bayes and 247.7 Ma from MLA chronograms) fall within the limits of these simulations.

**Figure 6:**
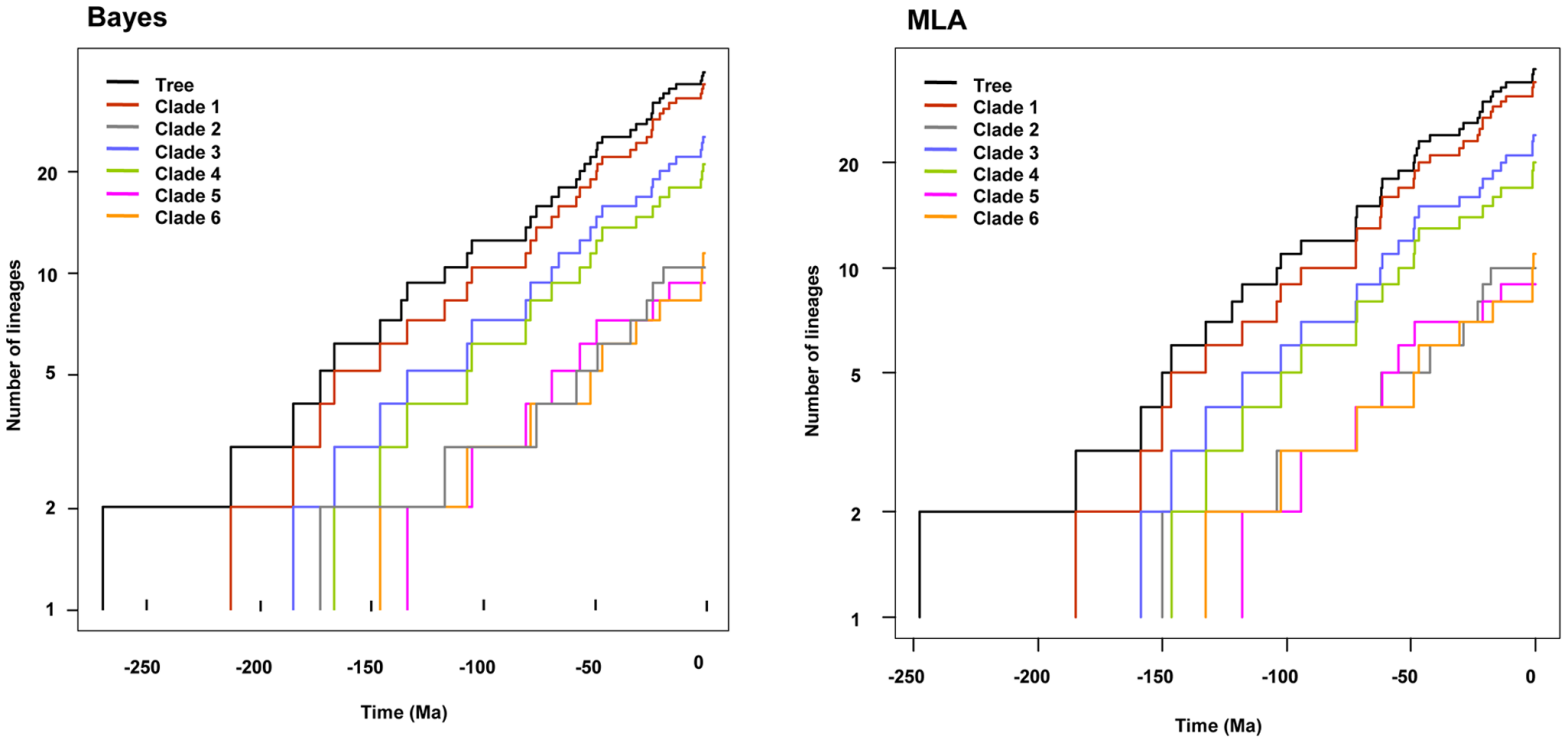
LTT plots for the major clades of *Aeromonas*. Log-lineage-through-time (LTT) plot for the six major clades inferred by Bayesian (left) and MLA (right) analysis.

Another important question to consider when verifying the suitability of a diversification model for our data is the determination of the diversification rate variation across lineages in the phylogeny. Figure 6 shows the LTT plots corresponding to the six major clades, which exhibit the same pattern of diversification as the entire tree, and each one fits well to a straight line parallel to that of the entire tree. In all cases the Yule model was selected by Rabosky’s ΔAIC_RC_ test as the model that best describes the data (Table 3). The range of diversification rates obtained for this analysis was 0.0107–0.0145 for the Bayes chronogram, and 0.0115–0.0154 for the MLA chronogram. These values are in good agreement with the rates obtained when considering all the analyzed species (0.0119 for Bayes and 0.0129 for MLA; Table 2). Thus, we can conclude that there is little or no among-lineage variation in diversification rates in our *Aeromonas* phylogeny.

**Table 3:** Diversification rates and model of speciation for the major clades of *Aeromonas.*

		Bayesian				MLA			
Clade^a^	N	ΔAIC_RC_	*P* value	Bestmodel	λ (ML) ± se	ΔAIC_RC_	*P* value	Bestmodel	λ (ML) ± se
1	34	−1.1976	0.6126	Yule	0.0132±0.0023	−1.2177	0.6031	Yule	0.0143±0.0018
2	10	−1.6910	0.6538	Yule	0.0109±0.0038	−1.5032	0.5467	Yule	0.0115±0.0088
3	24	−1.7612	0.7849	Yule	0.0135±0.0029	−1.1437	0.5377	Yule	0.0147±0.0022
4	20	−1.4868	0.6241	Yule	0.0136±0.0032	−0.5088	0.3563	Yule	0.0146±0.0025
5	9	0.5684	0.2242	Yule	0.0107±0.0040	1.1205	0.2543	Yule	0.0116±0.0031
6	11	0.3735	0.2253	Yule	0.0145±0.0048	1.6440	0.1270	Yule	0.0154±0.0053

a clade numbers appear in the MLA chronogram in Figure 2.

Clades 7 and 8 were not analysed due to their low number of species (Fig. 2).

Abbreviations: N, clade size; se, standard error.

**Figure 7:**
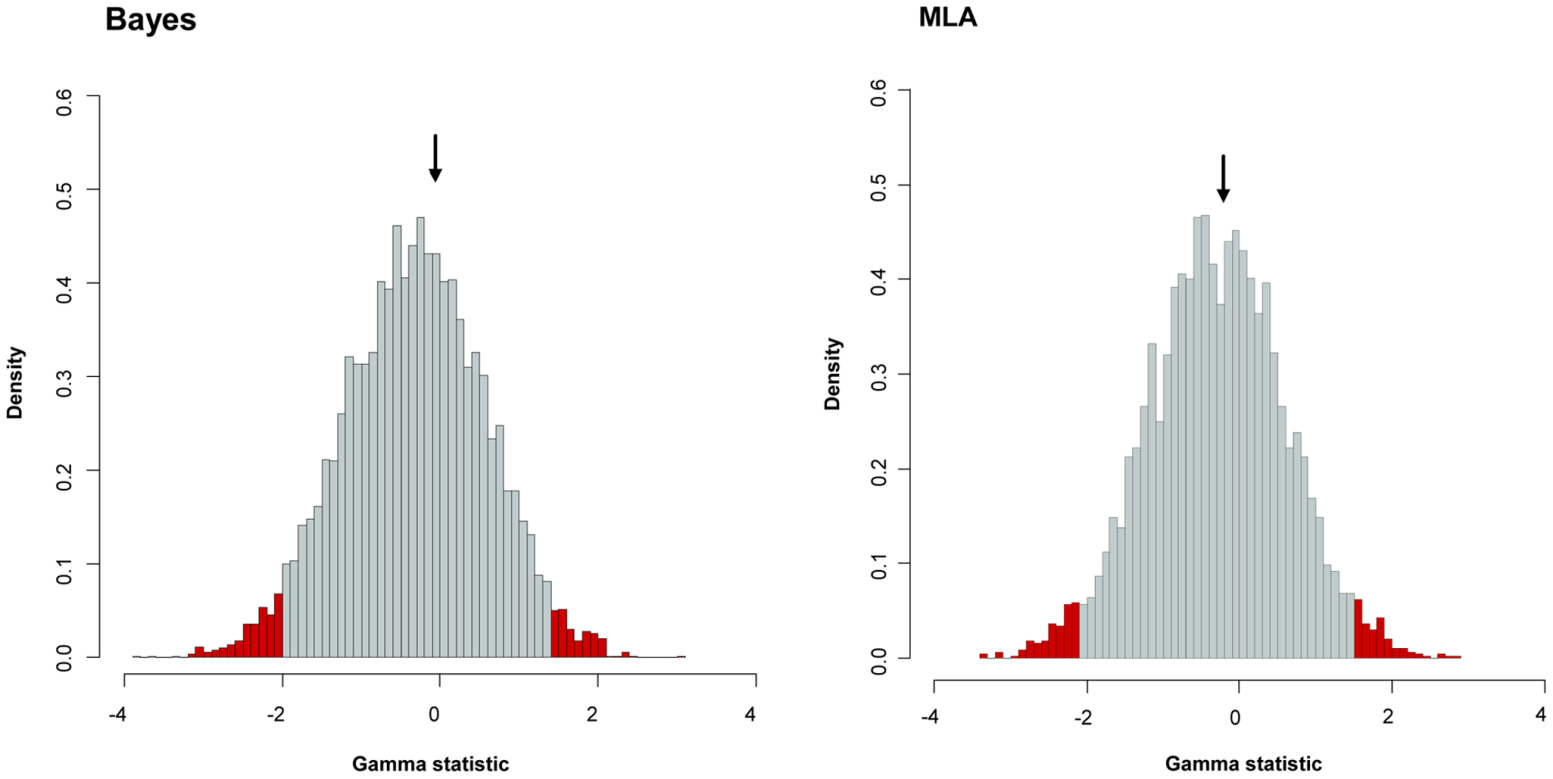
Gamma statistic distribution. Gamma statistic distribution was obtained by simulating 5,000 phylogenies under a Yule model using Bayes (left) and MLA (right) approaches. The arrows indicate the gamma value obtained from our sequences. Red bars indicate the 95% limits of the distribution.

The gamma statistic is a powerful tool to test the constancy of diversification rates, and is principally used for comparing models of decreasing speciation rate through time and constant-rate diversification [36], [37]. We thus obtained an estimated γ in both the Bayes (γ = -0.071) and MLA (γ = -0.225) chronograms. Although both γ values were negative, suggesting a possible deceleration of diversification rates through time, they were greater than those corresponding to critical values obtained by simulating 5,000 trees under a constant rate model (Bayes: γ = −2.053, and MLA: γ = −2.131) at a level of α = 0.05. Thus, a constant diversification rate had to be accepted for our phylogenies. In the case of the Bayesian chronogram, we were also able to compare the gamma statistic calculated from the posterior distribution of trees (mean = 0.046; 95% CI: −0.680–0.837), the values being within the limits of the simulated data for the Bayes chronogram (95% CI: −2.053–1.350) and MLA chronogram (95% CI: −2.131–1.394) (Fig. 7).

It is well known that LTT plot results are sensitive to incomplete taxon sampling [4], [61]. In order to discard the influence of incomplete sampling due to the likely existence of undescribed species, we performed a Monte Carlo Constant Rate (MCCR) test, assuming a possible total number of 50, 60 and 70 *Aeromonas* species (Table 4). The results obtained did not allow us to reject the hypothesis of a constant rate of diversification in neither the Bayes nor the MLA chronograms at a level of α* = *0.05. These results indicate that although we obtained negative γ values in both cases, they are not significantly negative when compared with the null model of the constant diversification rate, irrespective of the effect of taxon sampling.

**Table 4:** Monte Carlo Constant Rates (MCCR) test.

	Bayesian chronogram^a^			MLA chronogram^b^		
N^c^	Prob.^d^	Criticalvalue	*P* value	Prob.^d^	Criticalvalue	*P* value
50	0.8838	−1.9198	0.6151	0.8762	−1.8759	0.5409
60	0.9245	−2.2675	0.7345	0.9185	−2.2560	0.6788
70	0.9509	−2.4945	0.8314	0.9463	−2.5104	0.7904

a λ = 0.0119 and t* = *265.7 Ma.

b λ = 0.0129 and t = 247.7 Ma.

c hypothetical clade size.

d probability that *Aeromonas* has N or less species according to the Yule model.

The standardized Colless’ index value for both the Bayes and MLA chronograms was identical, *I_c_* = 0.1562, an unsurprising result considering that both chronograms have the same topology. This value did not differ significantly (*P*>0.3) from those calculated through the *I_c_* distribution obtained from 5,000 simulated phylogenies under a Yule model (95% CI ranging between 0.0781 and 0.2282 for both chronograms). Likewise, the number of cherries in both chronograms was 13. Both the normal approximation (*P*>0.6) and the comparison with the cherry distribution in 5,000 simulated trees (*P*>0.7) allowed us to accept the null hypothesis of a Yule model. The results obtained from the aforementioned statistical tests also support the hypothesis of a constant diversification in *Aeromonas*.

The mean substitution rate obtained from the Bayesian chronogram was 9.80×10^−4^ substitutions per site per Ma (se. = 2.53×10^−5^). This value almost fully coincides with that obtained through the MLA chronogram: 1.06×10^−3^ substitutions per site per Ma (se. = 2.53×10^−5^). Both mean substitution rates were obtained by regression analysis of branch lengths of both chronograms and those of the original maximum likelihood tree. As shown in the supplementary material (Fig. S1), there is a robust linear relationship between synonymous substitutions per synonymous site and sequence divergence in our concatenated sequences (slope = 2.8486; *R^2^* = 0.9947). This relationship allowed us to convert our estimates of substitution rates to synonymous substitutions per synonymous site per Ma. The values obtained were 2.79×10^−3^ for Bayesian and 3.02×10^−3^ for MLA, implying a silent substitution rate of 0.3% per Ma, which is slightly lower than the average silent substitution rate estimated for *E.coli* and *Salmonella* (0.45%), assuming that universally distributed proteins evolve at the same rate in enteric bacteria as in mammals [62], [63], and is similar to that observed in other protein coding genes in *Salmonella* and *Escherichia* [64].

In summary, the analysis of the LTT plots obtained with our phylogeny, the fit of the best model of diversification through maximum likelihood, the comparison with null models obtained by simulated trees, the gamma statistic of Pybus and Harvey and the tree imbalance tests all confirm that our phylogenetic trees are best explained by assuming a Yule model of constant diversification. The diversification rate of the genus *Aeromonas* ranges from 0.0119 to 0.0129 per Ma, depending on the dating method used (Bayesian or MLA). This constant rate remains virtually unchanged through time and across the different major clades of the phylogeny.

## Discussion

The explosion of molecular data in recent years has culminated in a vast accumulation of prokaryote genomic information. However, this huge amount of information has not been used to unveil the speciation mechanisms of prokaryotes nor to clarify the conflicting hypotheses on the prokaryote species concept [28], [65]–[68]. As a consequence, prokaryotes are still subject to far more controversy than their eukaryotic counterparts. Thus, understanding the evolution of biological diversity of prokaryotes remains a great challenge for biologists [22], [23], [69]. The issue is far from trivial because many problems of extreme importance to human society hinge on understanding prokaryotic diversity and how it will respond to change [69]. Although few studies of this type have been carried out [22]–[24], [69], in our opinion, knowledge of the diversification rate and pattern of a bacterial genus may be useful for understanding prokaryotic evolution [70].

In this work, we studied the phylogeny and diversification rates of *Aeromonas* by applying methods previously used with eukaryotic taxa. Assuming that the cohesion of major phylogenetic groups within the prokaryotes is due to vertical transmission and common ancestry rather than to preferential horizontal gene transfer (HGT), it is possible to construct robust phylogenies reflecting the evolutionary history of bacteria using a sufficient number of orthologous housekeeping (core) genes. In these phylogenies most bacterial species are delineable as discrete evolutionary lineages [71]–[73].

The foregoing does not exclude the existence of HGT, which in fact occurs and has important evolutionary consequences, but it is doubtful that HGT is the essence of modern genome phylogeny [72]. Besides ecological isolation, mechanisms of sexual isolation, such as the obstacles to DNA entry in bacterial cells or restriction endonuclease activity, can significantly reduce the effectivity of HGT [66]. Moreover, as demonstrated in *Salmonella*, *Streptococcus* and *Bacillus*, homologous recombination decays exponentially with sequence divergence, that is, a sequence divergence between two strains of 10% suppresses the recombination rate between them by a factor of about 100 [74], [75]. This suggests that most genes acquired by HGT were probably introduced only rarely and very early in the evolutionary history of these bacterial species [67].

### Phylogenetic Relationships of *Aeromonas* Species

Our phylogenetic analysis of the genus *Aeromonas* corroborates the monophyletic origin of this group of bacteria. The chronogram topology obtained from the Bayesian approach coincided fully with the MLA chronogram, confirming the robustness of our phylogeny (Fig. 2). The distribution of the main clades in our trees is in complete agreement with previous similar studies [76]–[78], although divergences occasionally appear in comparisons with phylogenies constructed with single genes [39]–[41]. Our study also provides further evidence for the existence of subspecies at the limit of being considered separate species, for example, *A. hydrophila* subsp. *dhakensis* [79], or for the relocation of what have been considered as new species, such as *A. culicicola* and *A. aquariorum* [26], [79], [80].

In the phylogeny we obtained, the different lineages that lead to the present species showed a mean pairwise divergence of 10% (ranging from 8.8 to 14.1%), a value that is enough to make recombination highly improbable, even if there was a relatively high recombination rate in *Aeromonas* [68]. Further evidence for this assumption was provided by an independent approach, a split decomposition analysis using SplitsTree4 software (Fig. S2). This analysis showed the absence of reticulated phylogenetic structures suggesting no evidence of detectable recombination in *Aeromonas*.

### *Aeromonas* Diversification Rates

Both MLA and Bayesian chronograms suggest that the divergence of the genus *Aeromonas* began at an indeterminate point between the Permian and Triassic periods and has continued exponentially until today (Fig. 2). Slight differences between the Bayesian (265.7 Ma) and MLA (247.7 Ma) ancestry estimates may be due to the relaxed clock method used to infer dates and the prior distribution for the divergence time of the calibration point in the Bayesian versus the minimum and maximum age constraints in the MLA method. Moreover, we are aware that the use of a single calibration point can be a source of uncertainty, which is very difficult to minimize in the absence of more reliable calibration data and the impossibility of accepting the hypothesis of a molecular clock evolution for our sequences. However, if a reliable phylogeny is obtained, as in our *Aeromonas* study, it is possible even with a single calibration point to make useful statements about bacterial divergence times [81]. The calibration point we have used, 140 Ma (120–160 Ma), for the divergence between *Escherichia* and *Salmonella* was proposed by Ochman and Wilson based on calibrated rates of ribosomal RNA divergence. This date roughly coincides with the appearance of the principal niche of *E. coli*, the mammalian intestine. A similar date of divergence for *E. coli* and *Salmonella* (100–130 Ma) was obtained when assuming that universally distributed proteins evolve at the same rate in enteric bacteria as in mammals [62] and a somewhat broader range of divergence times (57–176 Ma) was estimated based on biogeochemical evidence of cyanobacterial divergence [82].

The data obtained from both analyses (Bayes and MLA) were in good agreement although the estimations were obtained from two completely different approaches. Moreover, the MLA method, recently described by Paradis, requires far less computing than the Bayesian approach, yet gives more accurate results than the Penalized Likelihood method [21].

LTT plots, diversification tests and comparisons between simulated and empirical phylogenies give support to the hypothesis of a constant rate of cladogenesis in *Aeromonas* during all the Phanerozoic with no or an undetectable extinction rate (Tables 2 and 3; Fig. 2). The rate of diversification varies between 0.0119 and 0.0129 according to the method used for the analysis (Bayesian and MLA, respectively). Unfortunately, we can not compare our results with those of other authors, since, to our knowledge, no studies have been previously published on the diversification of an entire bacterial genus. Martin et al. [22] used sequences from ribosomal genes of a wide variety of prokaryotes obtained from alpine soils or databases to determine their diversification pattern, which in all cases proved to be constant over time, but without a quantitative estimation of the diversification rates. These results were remarkably homogeneous regardless of the bacterial group analyzed or the method used for constructing chronograms. More recently, Morlon et al. [24] used multilocus and genomic sequence data to determine the diversification rate of *Borrelia burgdorferi* sensu lato, a pathogenic intracellular bacterium. In this case, the pattern of diversification was not constant, with explosive radiations followed by rapid decreases in diversification rates. This different pattern of cladogenesis could be explained considering that *B. burgdorferi* is obligatorily associated with vertebrate and arthropod hosts, which may limit the gene flow between isolated populations and result in a type of diversification similar to that of eukaryotes.

Given the paucity of prokaryotic data, comparison with other bacterial taxa is impossible but comparison with available estimates of diversification rates for eukaryotic taxa may be insightful. *Aeromonas* appears to have a lower rate of diversification than other existing taxa. Our values are close to the minimal diversification rates (0.0162, 0.0092 and 0.0143, respectively) but far from the mean values (0.0753, 0.1859 and 0.0750, respectively) estimated for fish, birds and mammals [83]. This low rate of speciation does not seem related to the rate of substitution calculated from our sequences (0.3% per Ma), which is similar to that obtained for many species of both prokaryotes and eukaryotes [62]–[64].

Allopatric isolation is controversial in prokaryotes. As with many bacterial species, the majority of *Aeromonas* strains are isolated regularly in very different locations, virtually anywhere on the planet. In bacteria, genetic isolation would be achieved only, but not totally, by genetic (DNA) divergence. In more complex organisms (multicellular eukaryotes) the number of mechanisms leading to sympatric reproductive isolation increases considerably (ploidy, hybridization, reproductive behavior) and the number of genes in which one or several mutations can lead to a reproductive isolation is large.

**Figure 8:**
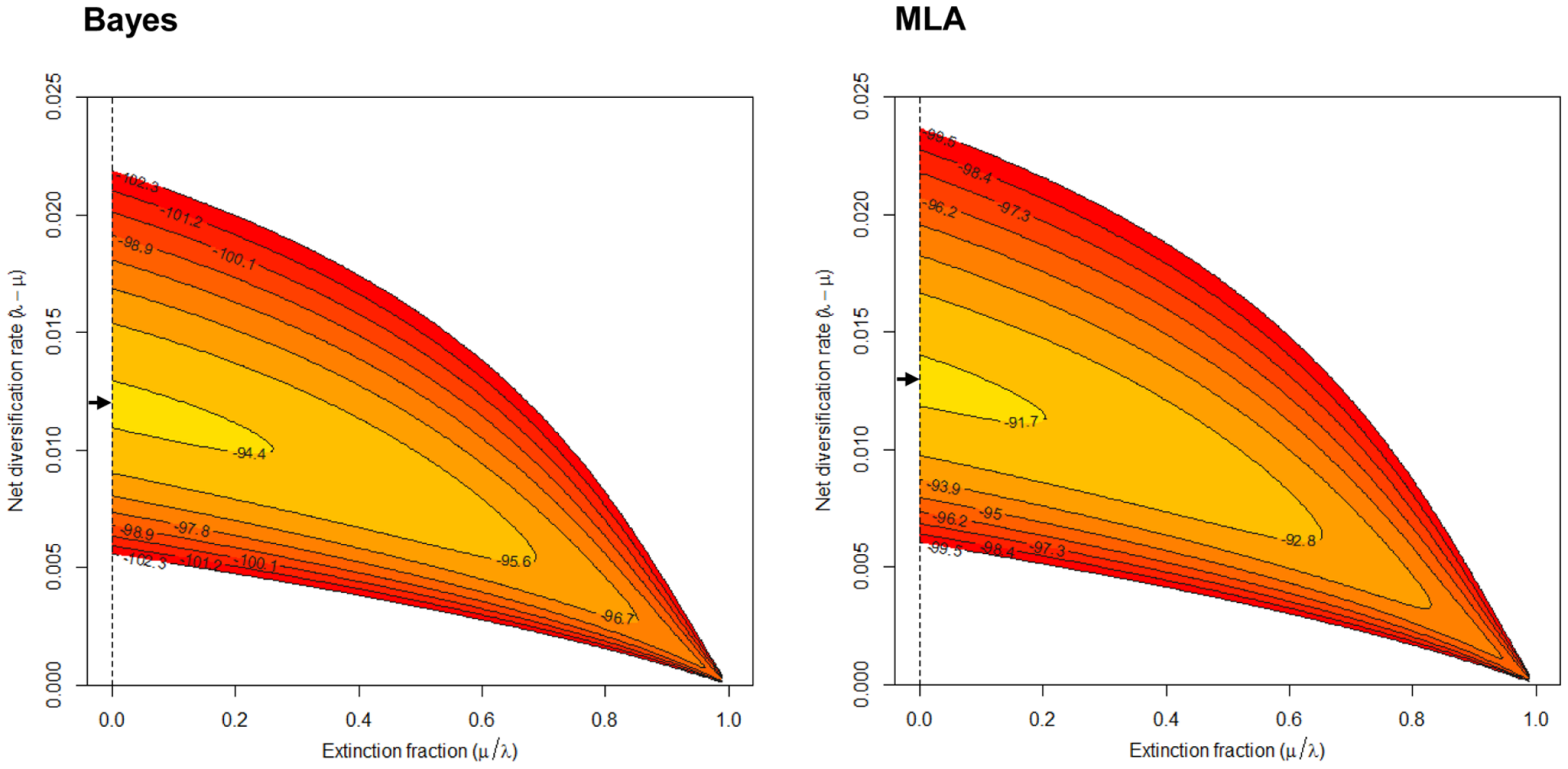
Contour plots of the likelihood surfaces. Contour plots of the likelihood surface were inferred from the relation between the net diversification rate (λ – μ) and extinction fraction (μ/λ) for the Bayes (left) and MLA (right) chronograms. Likelihoods were calculated using the R package LASER. The maximum likelihood estimates (i.e., the peak of the surface) are marked with an arrow.

The fact that our data fits a Yule model, a constant rate birth-death process with death rate μ = 0, in which each species evolves independently and produces new species at a constant rate λ, raises some questions. First of all, the parameters of this diversification model, the net diversification rate and the extinction fraction, have been determined by a maximum likelihood method. Generally accepted interpretations of this statistical method tell us that, for a fixed set of data (our sequences) and an underlying statistical model (the Yule model), the method of maximum likelihood selects the values of the model parameters that produce a distribution, giving the observed data the greatest probability. Figure 8 shows the contour plots of the log likelihood surface for our data. These plots reveal that although the maximum likelihood estimate of the extinction fraction (μ / λ) is zero, we cannot completely exclude the possibility that the extinction rate (μ) has a small but appreciable value.

To address this question, we first estimated a 95% confidence interval of the expected relative extinction fraction (*r* = μ / λ) by simulation of 5,000 phylogenies under the Yule model and obtained identical results from both Bayes and MLA approaches (0–0.62). On the basis of the divergence data from the basal node of the *Aeromonas* clade (247.7 Ma for Bayes and 265.7 Ma for MLA) and the observed diversity of 37 lineages, we used the estimates (0–0.62) to calculate the net diversification rate (*a* = λ – μ) for *r* = 0 (0.0135 for Bayes and 0.0146 for MLA) and *r* = 0.62 (0.0101 for Bayes and 0.0108 for MLA), using the Magallón and Sanderson approach [84] as implemented in the package LASER. These results suggest that even when assuming a relative extinction rate as high as 0.62, the net speciation rate remains reasonably close to the values obtained in our analysis (0.012 for Bayes and 0.013 for MLA).

Huge populations of prokaryotes are relatively immune to the extinction and founder effects experienced by larger, less abundant, organisms [85], [86]. Bacterial species may be considered as metapopulations (i.e. sets of connected subpopulations that are maximally inclusive and whose boundaries are set by evolutionary cohesive forces) that extend over time and that evolved separately from other species [65]–[67], [87]. Metapopulation models predict that the metapopulation will go extinct only if the ratio between the within-subpopulation extinction and colonization rates is greater than or equal to the availability of habitats for this species [88]. Although geographical barriers to microbial dispersal can be relatively common and physical isolation can play a certain role in microbial evolution, it is reasonable to assume that bacteria have essentially unlimited capacity for dispersal [89]. Bacterial size is on average in the micrometer range and passive dispersal can easily occur via a variety of mechanisms, including transport in the atmosphere, water currents, or transport on or within larger plants and animals and are more likely to be transported long distances [90]. Moreover, bacterial populations may be very large and have high growth rates under favorable environmental conditions, and adopt physiologically inactive states for extended periods of time and survive during unfavorable ones [91].

If we combine this high probability of dispersal with the fact that a free-living heterotrophic, facultatively anaerobic bacteria such as *Aeromonas* can be isolated from virtually every environmental niche, including aquatic habitats, soils, fresh and marine waters, plant surfaces, invertebrates, fish, reptiles, birds, and food [26], and environmental conditions (pH, temperature), the availability of potential habitats for such prokaryotes is astronomically large and therefore, the probability of extinction of the *Aeromonas* metapopulation is null or very low.

**Figure 9:**
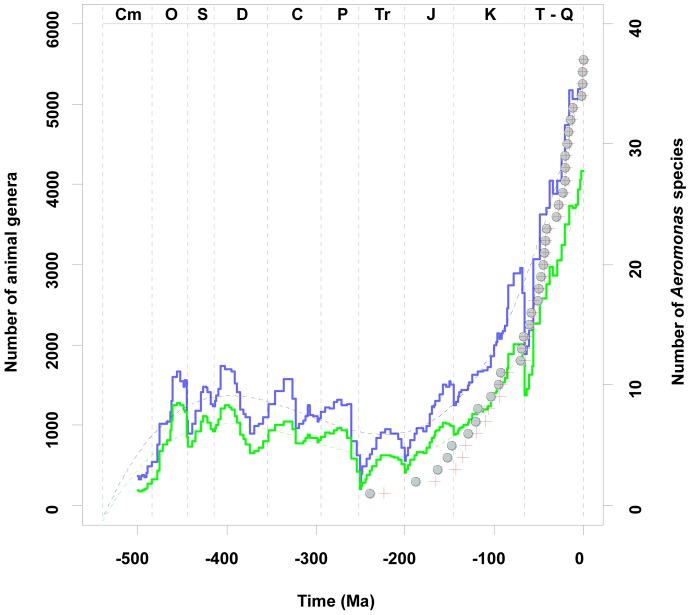
Diversification of *Aeromonas* compared with the number of animal genera versus time. Diversification of *Aeromonas* determined by Bayesian (circles) and MLA (crosses) approaches, compared with the number of animal genera versus time obtained from Sepkoski’s compendium, converted to the 2004 Geologic Time Scale [95], [96]. The blue line represents the total number of animal genera while the green line shows the same data with single occurrence and poorly dated genera removed. Dashed lines indicate polynomial fits to data. Abbreviations: Cm; Cambrian, O; Ordovician, S; Silurian, D; Devonian, C; Carboniferous, P; Permian, Tr; Triassic, J; Jurassic, K; Cretaceous, and T-Q; Tertiary-Quaternary.

As can be seen in Figure 9, since the Permian-Triassic, the diversification of the genus *Aeromonas* runs parallel to the increase of animal genera. This diversification seems to have begun after the Permian-Triassic extinction of approximately 251 million year ago, when more than 90% of marine and terrestrial life became extinct [92], possibly causing a return to an ancient world dominated by microorganisms [86].

**Figure 10:**
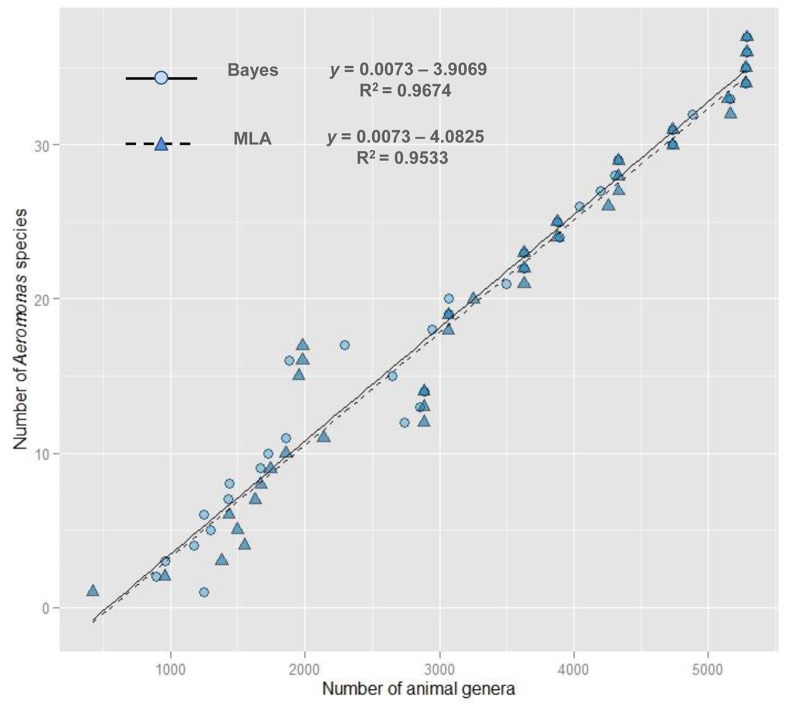
Regression plot between the number of animal genera and the number of *Aeromonas* species. The plot shows the number of animal genera obtained from Sepkoski’s data and the number of *Aeromonas* species in the last 250 Ma, applying both the Bayesian and the MLA approaches.

Analysis of the fossil record of microbes, fungi, plants and animals shows that the diversity of both marine and continental life, although interrupted by mass extinctions, has increased exponentially since the end of the Precambrian [93]. Fossil records also suggest that after the end-Permian extinction, eukaryotic life, primarily multicellular plants and animals, diversified at an exponential rate through most of the Mesozoic and Cenozoic [92]–[94]. This explosive proliferation of multicellular organisms and their decisive influence on the structure and function of modern ecosystems in the Phanerozoic provided a new universe of potential ecological niches and the corresponding evolutionary opportunities for the bacterial lineages of the Paleozoic and Precambrian [86], [92]. Moreover, phanerozoic plants and animals have changed the ancient biosphere over evolutionary time, modifying biogeochemical cycles that are now intimately linked to the capacity of multicellular organisms to translocate nutrients across mixing boundaries, forcing the diversification of microorganisms to the new trophic structures [86]. The existence of a good correlation between the number of animal genera (according to Sepkoski’s data) and the *Aeromonas* diversification in the last 250 Ma (Fig. 10) corroborates this idea.

In conclusion, our results suggest that the diversification of the genus *Aeromonas* began 248–266 Ma ago, remaining constant through time and across the different major clades of the phylogeny, and runs parallel to the exponential increase of animal genera after the Permian-Triassic extinction. Their speciation rate is significantly lower than that found for many eukaryotic taxa, although the absence of quantitative prokaryote data makes comparison almost impossible. Two particular features distinguish our work from previous studies: firstly, it is based on a robust phylogeny of all the species and subspecies of a bacterial genus, and secondly, it has used two proven methods to estimate the absolute speciation rate and the approximate date of origin of *Aeromonas*. Our results appear to confirm those of Martin et al. [22] regarding the constancy of the diversification rate in prokaryotes. Nevertheless, considerable more research is required on other bacterial genera to test if our results are comparable with complete phylogenies of other bacterial taxa, including pathogens and free-living bacteria.

## Supporting Information

Figure S1 **Regression plot of sequence divergence versus synonymous substitutions per site.**
(TIF)

Figure S2 **Split decomposition analysis.** We used the split decomposition method to infer the 37 *Aeromonas* strains relatedness based on the concatenated sequence of five genes. Node labels refer to strain names (listed in). The split was generated by SplitsTree4 (v 4.13.1; www-ab.informatik.uni-tuebingen.de/software/splitstree4; Huson DH and Bryant D (2006) Mol Biol Evol 23∶254–267).
(TIF)

Table S1 ***Aeromonas*** **strains and GenBank accession numbers of gene sequences used in this study.**
(DOC)
