# Supplementary figures and images for: Molecular Phylogenetics and Temporal Diversification in the Genus Aeromonas Based on the Sequences of Five Housekeeping Genes
Source: PLoS One. 2014 Feb 20;9(2):e88805. doi: 10.1371/journal.pone.0088805 (PMC3930666; doi:10.1371/journal.pone.0088805)

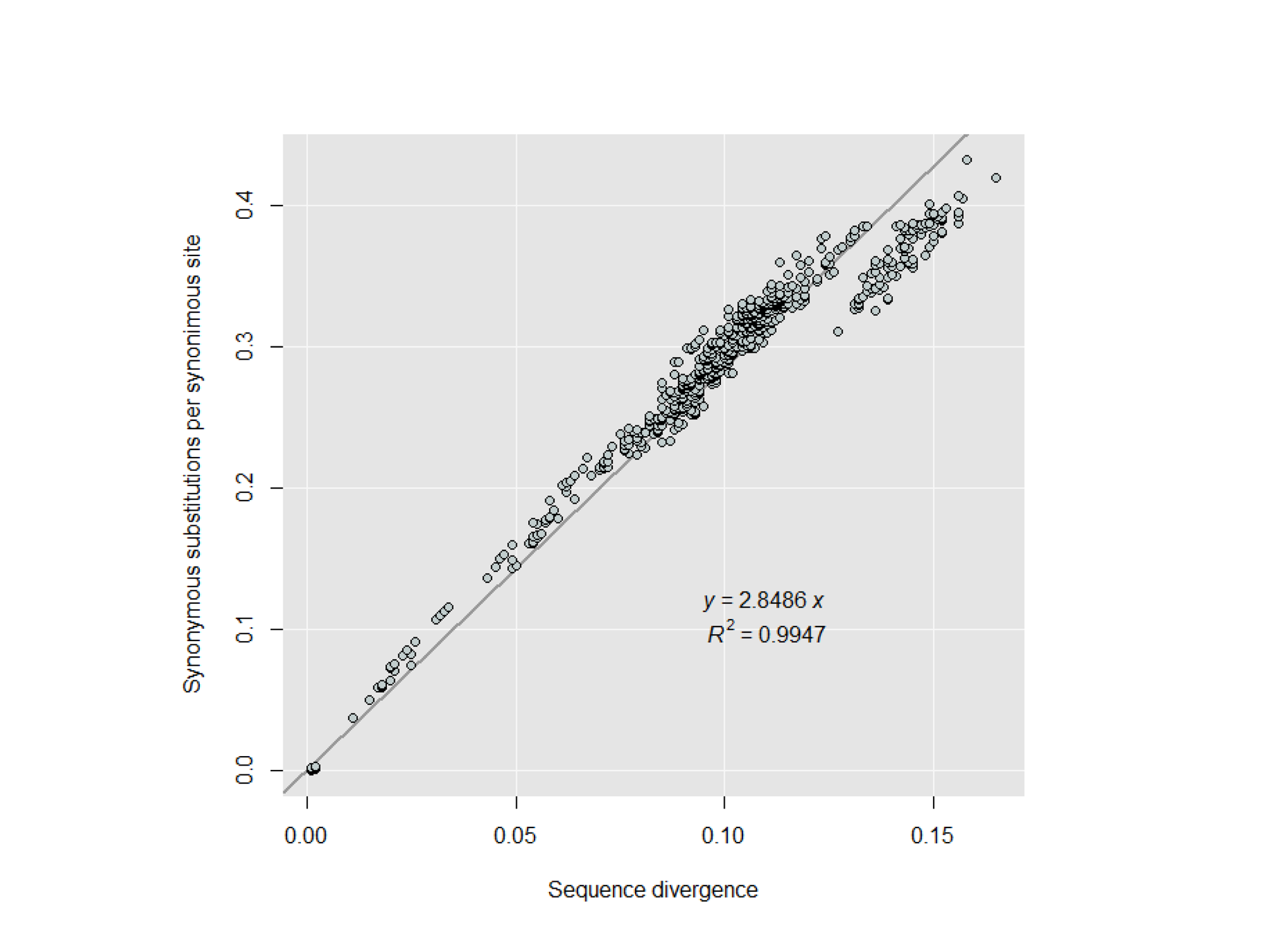

Supplement: Figure S1 — Regression plot of sequence divergence versus synonymous substitutions per site. (TIF) [file pone.0088805.s001.tif]

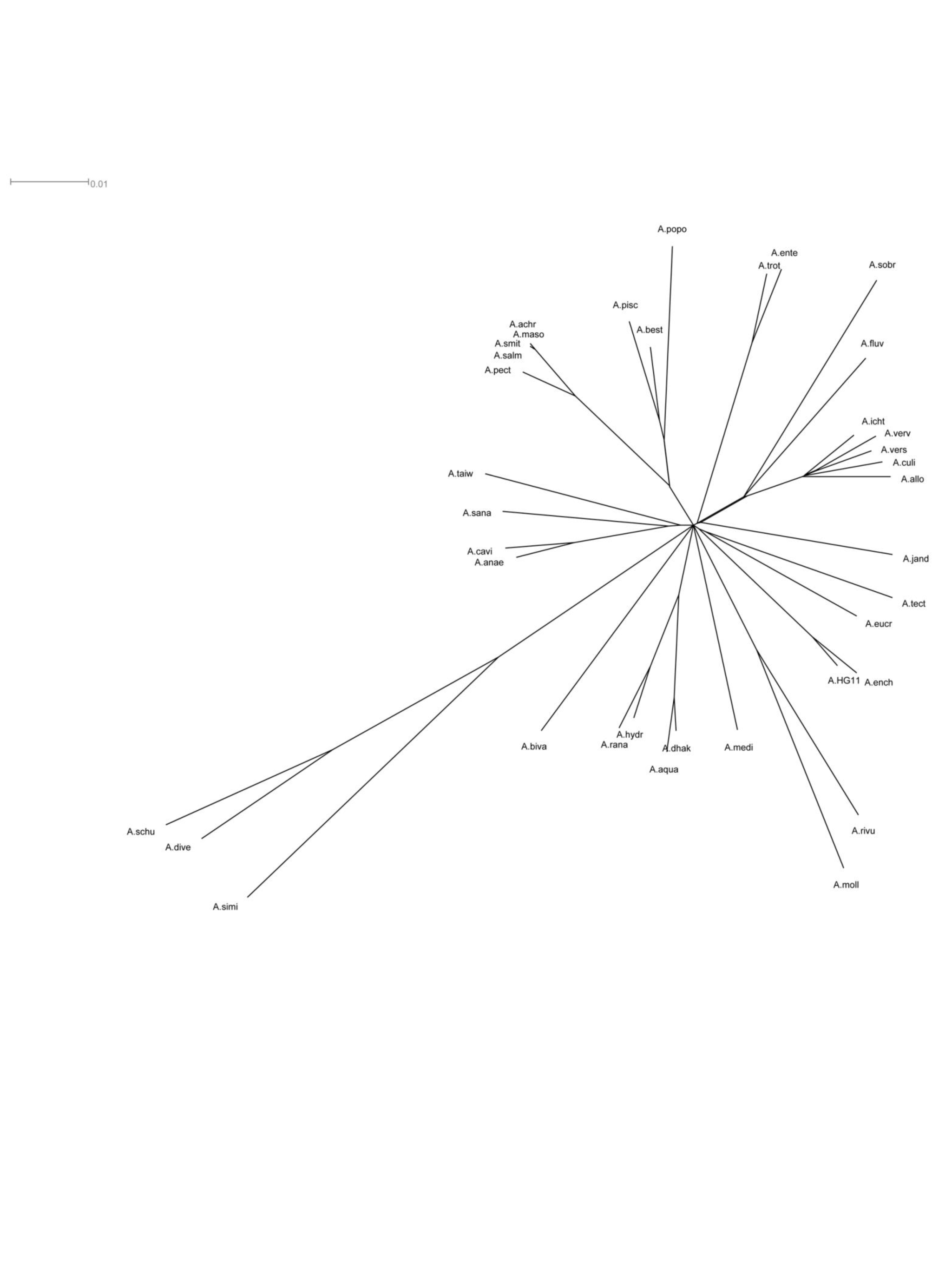

Supplement: Figure S2 — Split decomposition analysis. We used the split decomposition method to infer the 37 Aeromonas strains relatedness based on the concatenated sequence of five genes. Node labels refer to strain names (listed in). The split was generated by SplitsTree4 (v 4.13.1; www-ab.informatik.uni-tuebingen.de/software/splitstree4; Huson DH and Bryant D (2006) Mol Biol Evol 23∶254–267). (TIF) [file pone.0088805.s002.tif]
