# Supplementary material for: Molecular Phylogenetics and Temporal Diversification in the Genus Aeromonas Based on the Sequences of Five Housekeeping Genes
Source: PLoS One. 2014 Feb 20;9(2):e88805. doi: 10.1371/journal.pone.0088805 (PMC3930666; doi:10.1371/journal.pone.0088805)
Supplement: Table S1 — Aeromonas strains and GenBank accession numbers of gene sequences used in this study. (DOC) [file pone.0088805.s003.doc]

**Table S1. *Aeromonas* strains and GenBank accession numbers of gene sequences used in this study.**

| **Strain** | **GenBank accession number** | | | | |
| --- | --- | --- | --- | --- | --- |
|  | ***cpn60*** | ***dnaJ*** | ***gyrB*** | ***mdh*** | ***rpoD*** |
| *A. allosaccharophila* CECT 4199^T^ | EU306795* | AB280553 | AJ868385 | HM163292* | AY169348 |
| *A. aquariorum* MDC 47^T^ | FJ936120* | FJ936122* | EU268444 | HM163293* | FJ936132* |
| *A. bestiarum* CECT 4227^T^ | EU306796* | JN215529* | AJ868362 | HM163294* | JN215536* |
| *A. bivalvium* 868E^T^ | EU306799* | FJ936124* | EF465525* | HM163295* | EF465512* |
| *A. caviae* CECT 838^T^ | EU306800* | AB280555 | AJ868400 | HM163296* | AY169337 |
| *A. culicicola* CIP 107763^T^ | EU306840* | AB280556 | AJ868386 | HM163297* | DQ411505 |
| *A. diversa* CECT 4254^T^ | EU306835* | FJ936129* | GU062400* | HM163298* | AY169345 |
| *A. encheleia* CECT 4253 | EU306802* | AB280552 | AJ964951 | HM163300* | AY169343 |
| *A. encheleia* CECT 4342^T^ | EU306801* | AB280557 | AJ868375 | HM163299* | AY169346 |
| *A. enteropelogenes* CECT 4487^T^ | EU306837* | AB280558 | EF465526* | HM163301* | EF465508* |
| *A. eucrenophila* CECT 4224^T^ | EU306803* | AB280559 | AJ868378 | HM163302* | AY169339 |
| *A. fluvialis* 717^T^ | GU062398* | FJ603454 | FJ603455 | HM163303* | FJ603453 |
| *A. hydrophila* subsp. *anaerogenes* CECT 4221^T^ | JN215525* | FJ999991 | AM262164 | HM163304* | AY185587 |
| *A. hydrophila* subsp. *dhakensis* CECT 5744^T^ | EU306806* | JN215530* | AM262163 | HM163305* | EF465510* |
| *A. hydrophila* subsp. *hydrophila* CECT 839^T^ | EU306804* | JN215531* | AJ868394 | HM163306* | JN215537* |
| *A. hydrophila* subsp. *ranae* CIP 107985^T^ | EU306805* | AB280562 | AM262162 | HM163307* | EF465509* |
| *A. ichthiosmia* CECT 4486^T^ | EU306841* | AB280563 | EF465527* | HM163308* | AY169342 |
| *A. jandaei* CECT 4228^T^ | EU306807* | AB280564 | AJ868391 | HM163309* | AY169341 |
| *A. media* CECT 4232^T^ | EU306808* | AB280565 | AF417627 | HM163310* | AY169338 |
| *A. molluscorum* 848T^T^ | EU306811* | AB280566 | EF465521* | HM163311* | EF465515* |
| *A. piscicola* S1.2^T^ | GU062399* | JN215532* | FM999963 | HM163312* | JN215538* |
| *A. popoffii* LMG 17541^T^ | EU306814* | AB280567 | AJ868372 | HM163313* | AY169347 |
| *A. rivuli* CECT 7518^T^ | JN215526* | FJ969432 | FJ969434 | JN215542* | FJ969433 |
| *A. salmonicida* subsp. *achromogenes* LMG 14900^T^ | EU306824* | AB280568 | AM262161 | HM163314* | JN215539* |
| *A. salmonicida* subsp. *masoucida* CECT 896^T^ | EU306825* | AB280569 | AM262160 | HM163315* | AY169330 |
| *A. salmonicida* subsp. *pectinolytica* CECT 5752^T^ | EU306827* | JN215533* | JN215535* | HM163316* | JN215540* |
| *A. salmonicida* subsp. *salmonicida* CECT 894^T^ | EU306828* | JN215534* | AY294485 | HM163317* | JN215541* |
| *A. salmonicida* subsp. *smithia* CECT 5179^T^ | EU306829* | AB280572 | AM262159 | HM163318* | AY169331 |
| *A. sanarellii* A2-67^T^ | JN215527* | FJ807279 | FJ807277 | HM163319* | FJ472929 |
| *A. schubertii* CIP 103437^T^ | EU306830* | AB280574 | AJ868402 | HM163320* | AY169336 |
| *A. simiae* CIP 107798^T^ | EU306833* | AB280573 | AJ632224 | HM163321* | DQ411508 |
| *A. sobria* CECT 4245^T^ | EU306834* | AB280575 | AF417631 | HM163322* | AY169340 |
| *A. taiwanensis* A2-50^T^ | JN215528* | FJ807270 | FJ807272 | HM163323* | FJ472928 |
| *A. tecta* MDC 91^T^ | FJ936121* | FJ936130* | AJ964952 | HM163324* | FJ936133* |
| *A. trota* CECT 4255^T^ | EU306836* | AB280576 | AJ868392 | HM163325* | AY169344 |
| *A. veronii* bv. Sobria CECT 4246^T^ | EU306839* | AB280578 | AY987514 | HM163326* | AY169333 |
| *A. veronii* bv. Veronii CECT 4257^T^ | EU306838* | AB280577 | AF417626 | HM163327* | AY127862 |

* Sequence data obtained by our research group

*A. culicicola* is later heterotypic synonym of *A. veronii* and *A. ichthiosmia* is synonym of *A. veronii*.

Abbreviations: CECT, Spanish Type Culture Collection; CIP, Collection de l’Institut Pasteur; LMG, Belgian Co-ordinated Collections of Microorganisms; T, type strain.
